# Molecular Epidemiology of Highly Diffusive DNA Viruses in Dogs and Cats from Romania

**DOI:** 10.3390/ani15243620

**Published:** 2025-12-16

**Authors:** Andrea Balboni, Lorenza Urbani, Mihaela Niculae, Cosmin Muresan, Martina Magliocca, Veronica Facile, Erika Esposito, Alessia Terrusi, Laura Gallina, Mara Battilani

**Affiliations:** 1 Department of Veterinary Medical Sciences, Alma Mater Studiorum—University of Bologna, Ozzano dell’Emilia, 40064 Bologna, Italy; a.balboni@unibo.it (A.B.); lorenza.urbani2@unibo.it (L.U.); martina.magliocca2@unibo.it (M.M.); veronica.facile2@unibo.it (V.F.); erika.esposito6@unibo.it (E.E.); alessia.terrusi2@unibo.it (A.T.); laura.gallina@unibo.it (L.G.); 2 Department of Infectious Diseases, University of Agricultural Sciences and Veterinary Medicine, 400372 Cluj-Napoca, Romania; mihaela.niculae@usamvcluj.ro; 3 Department of Surgery, Anesthesia and Intensive Care, University of Agricultural Sciences and Veterinary Medicine, 400372 Cluj-Napoca, Romania; cosmin.muresan@usamvcluj.ro

**Keywords:** canine adenovirus, canine circovirus, canine parvovirus, domestic carnivores, Europe, feline panleukopenia virus, molecular detection, viral diversity, virome

## Abstract

*Protoparvovirus carnivoran 1* (PPVC-1), Canine adenovirus type 1 and 2 (CAdV-1 and CAdV-2), and Canine circovirus (CanineCV) are highly diffusive viruses that affect domestic and wild carnivores worldwide, yet limited data are available on their circulation in Eastern European countries. In this retrospective study, the presence of these DNA viruses was investigated using molecular assays on fecal samples from 89 companion animals (56 dogs and 33 cats) collected in Romania between 2019 and 2021. The pathogens identified were analyzed genetically. Overall, 36/56 (64.3%) dogs and 5/33 (15.2%) cats tested positive for PPVC-1, 1/56 (1.8%) dogs for CAdV-1 and CAdV-2, and 15/56 (26.8%) dogs for CanineCV. In total, 40/56 (71.4%) dogs were positive for at least one of the screened pathogens. Novel findings in dogs included the frequent detection of canine parvovirus type 2c of Asian origin (Asian-like CPV-2c) and the first genetic data on CAdV-1 and CanineCV circulating in Romania. This study provides new insights into the epidemiology of DNA viruses in dogs and cats from Romania and highlights the need for ongoing monitoring of circulating pathogens to safeguard animal health, prevent outbreaks and limit potential transboundary spread.

## Simple Summary

In this study, fecal samples from 56 dogs and 33 cats collected in Romania between 2019 and 2021 were tested to investigate the presence of important viral pathogens affecting domestic and wild carnivores worldwide, including *Protoparvovirus carnivoran 1* (PPVC-1), Canine adenovirus type 1 and 2 (CAdV-1 and CAdV-2), and Canine circovirus (CanineCV). PPVC-1 was the most frequently detected virus, followed by CanineCV and CAdV. Sequencing results showed that most of the dogs were positive for the canine parvovirus type 2c variant, with amino acid residues in the deduced VP2 protein (324Ile and 370Arg) that are distinctive for “Asian-like” CPV-2c strains. One dog was co-infected with the original CPV-2 and feline panleukopenia virus. A single CAdV-1 was detected, and its molecular profile suggested potential temporal stability. Most of the identified CanineCV were genetically identical, except for one virus that showed genetic divergence and clustered phylogenetically with viruses typically reported in foxes, suggesting possible interspecies transmission. When interpreted in light of potentially limited vaccination coverage and ongoing companion animal trade practices, these findings highlight the importance of sustained pathogen monitoring and strengthened preventive measures to mitigate the risk of infectious disease dissemination.

## 1. Introduction

*Protoparvovirus carnivoran 1* (PPVC-1), Canine adenovirus type 1 and 2 (CAdV-1 and CAdV-2), and Canine circovirus (CanineCV) are highly diffusive DNA viral pathogens that affect domestic and wild carnivores worldwide. Assessing their distribution in susceptible hosts and their genetic characteristics is essential for understanding viral circulation and for implementing appropriate measures to prevent infections and associated diseases.

PPVC-1, belonging to the family *Parvoviridae* [1,2], includes two major viral pathogens responsible for severe acute gastroenteritis and immunosuppression in domestic and wild carnivores, often leading to high mortality in young animals [3], namely feline panleukopenia virus (FPV) and canine parvovirus type 2 (CPV-2) [4]. In Europe, the presence of these viruses in companion animals is well documented, with numerous reports describing FPV and CPV-2 as highly contagious pathogens that frequently cause serious and often fatal infections, especially in unvaccinated young animals [5,6,7]. All three CPV-2 antigenic variants (CPV-2a, CPV-2b, and CPV-2c) have been detected across Europe, with notable geographical variations in their distribution. In particular, CPV-2a predominates in most countries, except in Ireland and the UK, where CPV-2b is most prevalent [8]. CPV-2c was identified in several European countries [9], and it appears to be the predominant variant in Portugal, Poland, Romania and Southern Italy (Sicily) [10,11,12,13,14,15].

CAdV, a member of the *Adenoviridae* family [1], includes CAdV-1 and CAdV-2, the etiological agent of infectious canine hepatitis (ICH) and infectious tracheobronchitis in dogs [16], respectively. CAdV-1 infection has been reported worldwide [17]. In Europe, the disease has been linked to puppy trade from shelters in Eastern European countries and to viral circulation in wildlife populations [17]. Although primarily associated with upper respiratory tract infection, CAdV-2 has also been frequently detected in feces or internal organs of dogs [18,19]. The recommended vaccination of dogs with modified live CAdV-2 confers cross-protection and has significantly reduced CAdV-1 circulation in domestic dogs [16], but unvaccinated dogs in rural areas may still serve as a source of infection.

CanineCV, a member of the *Circoviridae* family [1,20], has been associated with vasculitis, hemorrhage and enteritis in dogs [21,22,23,24,25,26], as well as encephalitis in foxes [27], and has also been reported in cats [28]. Since its first identification in dogs from the USA in 2011 [29], CanineCV has been reported worldwide [23,24,25,26,30,31,32,33,34,35,36], and molecular data suggest that it may have circulated among carnivorous populations long before its initial discovery [37].

Although canine and feline parvovirosis are common infectious diseases in Romania, only a few studies have been published on CPV infection in dogs and FPV infection in cats [11,38], and no data are available on CAdV and CanineCV in dogs from this region. The aim of this study was to investigate the presence of PPVC-1, CAdV and CanineCV in fecal samples of dogs and cats from Romania in order to explore the virome of domestic carnivores, evaluate viral diversity, and provide new epidemiological data on these DNA viruses in Eastern Europe.

## 2. Materials and Methods

### 2.1. Study Design and Samples

In this retrospective study, the presence of PPVC-1, CAdV, and CanineCV was investigated in fecal samples of 89 companion animals (56 dogs and 33 cats) from Romania, collected between 2019 and 2021. The fecal samples consisted of surplus clinical material collected by veterinary practitioners for diagnostic purposes and used retrospectively for this study. Samples were stored at −20 °C until analysis. The dog population included: (i) 30 owned dogs, of which 20 were clinically healthy and 10 had acute gastroenteritis and were previously tested positive for CPV-2 DNA in a study by Balboni et al. [11]; (ii) five stray puppies rescued and housed in a private shelter, all showing clinical signs of acute gastroenteritis; and (iii) 21 clinically healthy dogs from a private shelter. The cat population included 26 clinically healthy and 7 sick stray animals. Signalment (sex, age, breed), vaccination status, and clinical data of the enrolled dogs and cats are available in AMSActa UNIBO at https://doi.org/10.6092/unibo/amsacta/8560 (accessed on 21 October 2025). The presence of PPVC-1, CAdV, and CanineCV DNA was assessed using quantitative molecular assays. Informative genes or the complete genome of the identified viruses were subsequently amplified, sequenced, and analyzed.

### 2.2. Detection of Protoparvovirus carnivoran 1, Canine Adenovirus, and Canine Circovirus DNA

DNA extraction from fecal samples was performed using the NucleoSpin Tissue Kit (Macherey-Nagel, Düren, Germany) according to the manufacturer’s instructions. Extracted DNA was stored at −20 °C until use.

The detection of PPVC-1, CAdV and CanineCV DNA was performed using three SYBR Green real-time PCR (qPCR) assays. All reactions were carried out in a total volume of 20 µL using the PowerUp SYBR Green Master Mix (Thermo Fisher Scientific, Life Technologies, Carlsbad, CA, USA), following the manufacturer’s instructions, and run on a StepOnePlus Real-Time PCR System (Thermo Fisher Scientific, Life Technologies, Carlsbad, CA, USA).

PPVC-1 DNA was investigated in fecal samples from 56 dogs and 33 cats using a qPCR assay targeting a 99-nucleotide (nt) fragment of the VP2 capsid protein gene [39]. CAdV DNA was investigated in fecal samples from 56 dogs using a qPCR assay targeting a 166-nt fragment of the E3 gene, which can discriminate CAdV-1 and CAdV-2 based on melting curve analysis [40]. CanineCV DNA was investigated in fecal samples from 56 dogs and 33 cats using a qPCR assay targeting a 132-nt fragment within the intergenic region (IR) between the two major ORFs [41]. The thermal cycling conditions for all assays consisted of an initial denaturation at 95 °C for 5 min, followed by 45 cycles of 95 °C for 15 s and 60 °C for 1 min. At the end of each run, a melting curve analysis was performed by continuously increasing the temperature from 55 °C to 98 °C to assess the reaction specificity. Characteristic melting temperatures were approximately 81–82 °C for PPVC-1, 73 °C for CAdV-1, 80 °C for CAdV-2, and 93 °C for CanineCV. Absolute quantification of viral DNA copy number was performed using the standard curve method. Serial 10-fold dilutions of a plasmid (pCR4 plasmid; Thermo Fisher Scientific, Life Technologies, Carlsbad, CA, USA) containing one copy of the corresponding target sequence served as external standards. Standard curves were generated by plotting threshold cycle values against plasmid copy number. Limits of detection (LOD) were determined as the highest plasmid dilution consistently amplified with good reproducibility and were established as 1 copy/µL for PPVC-1, 10 copies/µL for CAdV-1 and CAdV-2, and 5 copies/µL for CanineCV [39,40,41]. All DNA samples and standards were analyzed in duplicate. A no-template control, consisting of molecular-grade ultrapure water, was included in the analysis. An extraction blank was included in each DNA extraction batch and tested by qPCRs. Reactions were considered valid when target amplification was obtained in all the standard plasmid dilutions down to the LOD and no target amplification occurred in the no-template control. Samples exhibiting an exponential increase in fluorescence, a target DNA quantity greater than or equal to the LOD, and a specific melting peak in both replicates were considered positive, provided that the extraction blank was negative. Quantification of target DNA in positive samples was based on the mean value obtained from the two replicates.

### 2.3. Genetic Characterization of the Viruses Identified

The PPVC-1, CAdV, and CanineCV identified by qPCR screening were genetically characterized using a combination of end-point PCR assays, nucleotide sequencing, and bioinformatics analysis. Each PCR reaction was carried out in a total volume of 50 µL using the Phusion Hot Start II DNA Polymerase (Thermo Fisher Scientific, Life Technologies, Carlsbad, CA, USA), which contains a high-fidelity DNA polymerase, according to the manufacturer’s instructions. A no-template control, consisting of molecular-grade water, was included in the analysis.

For the identified PPVC-1, a 569-nt fragment of the VP2 gene was amplified using a heminested PCR, as described by Balboni et al. [39]. Canine parvovirus 2a (CPV-2a) 791/2014 (MK348096) [12] was used as the positive control.

For the identified CAdV, complete *hexon* and *fiber* genes were amplified using two PCR assays, as previously reported [42]. Amplicons of 2813 nt were generated for the *hexon* gene of both CAdV-1 and CAdV-2 [42]; while *fiber* gene was 1787 nts for the CAdV-1 and 1748 nts for CAdV-2 [42]. CAdV-1 417-2013-L (KP840546 and KP840547) [42] was used as the positive control.

For the identified CanineCV, the complete genome was amplified using a combination of rolling circle amplification (RCA) and PCR [41]. Initially, RCA was carried out using the TempliPhi 100 amplification kit (GE Healthcare, Chicago, IL, USA) to increase the circular DNA content. Subsequently, viral DNA was amplified using two PCR assays, following De Arcangeli et al. [41]. CanineCV 73/2017 (MT180081) [37] was used as the positive control.

PCR products (5 μL) were separated by electrophoresis on a 1.0% agarose gel prepared in 1 X Tris-acetate EDTA (TAE) buffer and visualized under UV light after staining with Midori Green Advance DNA Stain (Nippon Genetics, Düren, Germany). Samples were considered positive when an amplicon of the expected size was observed and the corresponding positive and no-template controls produced a positive and negative result, respectively. Positive amplicons were purified using the QIAquick PCR Purification Kit (QIAGEN, Hilden, Germany) and subjected to Sanger sequencing (BioFab Research, Rome, Italy), using both forward and reverse primers.

Nucleotide sequences obtained from one identified PPVC-1 (lab ID: 1356/2021) showed an unusually high number of ambiguities, with multiple chromatogram peaks suggesting a mixed viral population. To resolve this ambiguous sequencing result, the amplicon obtained for the PPVC-1 identified in dog 1356/2021 was re-sequenced after cloning into the pCR 4/TOPO vector, using the pCR 4-TOPO TA kit (Life Technologies, Carlsbad, CA, USA), and transforming it into *Escherichia coli* DH5α-competent cells. Specifically, all the *E. coli* colonies grown on Luria–Bertani (LB)-agar plates (Invitrogen, Thermo Fisher Scientific, Life Technologies, Carlsbad, CA, USA) supplemented with ampicillin (Gibco, Thermo Fisher Scientific, Life Technologies, Carlsbad, CA, USA) were picked, plasmids were extracted using the PureLink Quick Plasmid Miniprep (Life Technologies, Carlsbad, CA, USA), and each recombinant clone was sequenced individually to differentiate viral variants.

The obtained sequences were assembled, analyzed using the BLAST web interface (https://blast.ncbi.nlm.nih.gov/Blast.cgi, accessed on 25 February 2025), and aligned with reference sequences from the GenBank database (https://www.ncbi.nlm.nih.gov/genbank/, accessed on 25 February 2025) using the ClustalW algorithm implemented in BioEdit software version 7.2.5 (Tom Hall, Ibis Biosciences, Carlsbad, CA, USA). Nucleotide sequences were translated into amino acid sequences. Potential recombination events were assessed using the Recombinant Detection Program (RDP) version 4.101 [43]. Phylogeny was carried out using MEGA 11 software version 11.0.13 [44]. The optimal nucleotide substitution model for each dataset was selected using the Find Best DNA/Protein Model function. A Neighbour-Joining phylogenetic tree of the partial PPVC-1 VP2 gene was constructed using the Tamura 3-parameter model with gamma distribution. Maximum Likelihood phylogenetic trees were generated for the concatenated *hexon* and *fiber* genes of CAdV using the Hasegawa–Kishino–Yano model with gamma distribution and invariable sites, and for the complete genome of CanineCV using the General Time Reversible model with gamma distribution and invariable sites. Bootstrap analysis (1000 replicates) was performed to assess the robustness of the phylogenetic tree.

### 2.4. Statistical Analysis

Data were evaluated using standard descriptive statistics and are presented as median and range. Categorical variables were analyzed using Fisher’s exact test. Statistical significance was defined as *p* < 0.05. All analyses were performed using MedCalc Statistical Software version 19.5.1 (Ostend, Belgium).

## 3. Results

### 3.1. Molecular Detection of DNA Viruses

In Table 1, the signalment data, vaccination status, clinical presentation, and frequency of viral infection in tested animals are reported. Thirty-six out of 56 (64.3%) dogs and 5/33 (15.2%) cats tested positive for PPVC-1 DNA (Table 1). The overall median quantity of PPVC-1 DNA was 2.2 × 10^3^ copies of target DNA per microliter of extracted DNA (copies/μL) (range: 1.3 × 10^0^–1.5 × 10^10^) in dogs and 3.2 × 10^1^ copies/μL (range: 1.6 × 10^1^–2.2 × 10^3^) in cats. One out of 56 (1.8%) dogs tested positive for CAdV-1 and CAdV-2 DNA (Table 1), with 2.6 × 10^3^ copies/μL of CAdV DNA. Fifteen out of 56 (26.8%) dogs and 0/33 (0%) cats tested positive for CanineCV DNA (Table 1), with an overall median quantity of 2.6 × 10^2^ copies/μL (range: 6.3 × 10^0^–9.8 × 10^4^) of viral DNA in dogs. In total, 40/56 (71.4%) dogs tested positive for at least one of the screened viruses: 28/40 (70%) were positive for a single pathogen, while 12/40 (30%) were co-infected. Among the co-infected dogs, eleven were positive for PPVC-1 and CanineCV, and one was positive for PPVC-1, CAdV-1 and CAdV-2. The latter was a four-month-old owned female Siberian Husky with acute gastroenteritis and an incomplete vaccination protocol. The eleven dogs positive for both PPVC-1 and CanineCV were mixed breed clinically healthy animals from a private shelter, with a median age of six months (range: 2–18 months). Eight out of these 11 (72.7%) dogs were male and 3/11 (27.3%) were female; 8/11 (72.7%) were adequately vaccinated, 2/11 (18.2%) were unvaccinated, and 1/11 (9.1%) had an incomplete vaccination protocol.

Viral infection in tested dogs was significantly associated with age (*p* = 0.0173), due to a higher frequency of PPVC-1 infection in young dogs (*p* = 0.0249); with vaccination status (*p* = 0.0031), due to a higher frequency of PPVC-1 infection in unvaccinated or improperly vaccinated dogs (*p* = 0.0017); and with clinical status, due to a higher frequency of PPVC-1 infection in dogs with acute gastroenteritis (*p* = 0.0004) and of CanineCV infection in healthy dogs (*p* = 0.0054) (Table 1). The habitat in which the dogs lived was not significantly associated with DNA virus infection overall (*p* = 0.2357), except for CanineCV (*p* = 0.0001), which was detected almost exclusively in shelter dogs rather than owned dogs (14/26 and 1/30, respectively). No statistical association was found between PPVC-1 DNA detection and the signalment data, vaccination status, or clinical presentation of the tested cats (Table 1).

### 3.2. Genetic Characterization of the Viruses Identified

A partial PPVC-1 VP2 gene nucleotide sequence of 532 nts in length (from nt 883 to 1414 of the CPV-2c reference strain 239/08, GenBank ID: FJ005251) was obtained from 11 dogs in this study. Analysis of the deduced amino acid residues at critical positions enabled the identification of ten CPV type 2c (CPV-2c), based on the presence of glutamate at position 426 (codon GAA). Nine out of these ten CPV-2c sequences (lab IDs: 1342/2021, 1345/2021, 1349/2021, 1355/2021, 1386/2021, 1387/2021, 1388/2021, 1389/2021 and 1390/2021) showed complete nucleotide identity with one another and with CPV-2c sequences previously obtained from ten additional dogs included in this study (lab IDs: 157/2019 through 166/2019) and reported by Balboni et al. [11]. The remaining CPV-2c sequence (lab ID: 1360/2021) showed a synonymous substitution at position 1329 (C > T) of the VP2 gene. The putative VP2 amino acid sequence of all CPV-2c identified in this study had the two distinctive amino acid residues 324-isoleucine (Ile) and 370-arginine (Arg). For the remaining PPVC-1 identified in the eleventh dog (lab ID: 1356/2021), the partial VP2 gene amplicon was cloned, and nucleotide sequences were obtained for three recombinant clones. Two clones (lab IDs: 1356/2021-cl02 and 1356/2021-cl04) were identical and corresponded to the original CPV-2 type characterized by the distinctive amino acid residues 375-asparagine (Asn), 386-lysine (Lys) and 418-threonine (Thr). The third clone (lab ID: 1356/2021-cl08) corresponded to FPV.

In cats, a partial PPVC-1 VP2 gene nucleotide sequence of 532 nts in length (from nt 883 to 1414 of the FPV reference strain 443/07, GenBank ID EU498718) was obtained from four subjects (lab IDs: 1332/2021, 1367/2021, 1369/2021, and 1370/2021). Analysis of the deduced amino acid residues identified all four sequences as FPV. Three out of them (1367/2021, 1369/2021, and 1370/2021) were identical to each other and also matched the FPV sequence detected in dog 1356/2021 (1356/2021-cl08). The FPV sequence 1332/2021 exhibited a synonymous substitution at position 1041 (A > G) of the VP2 gene.

**Figure 1:**
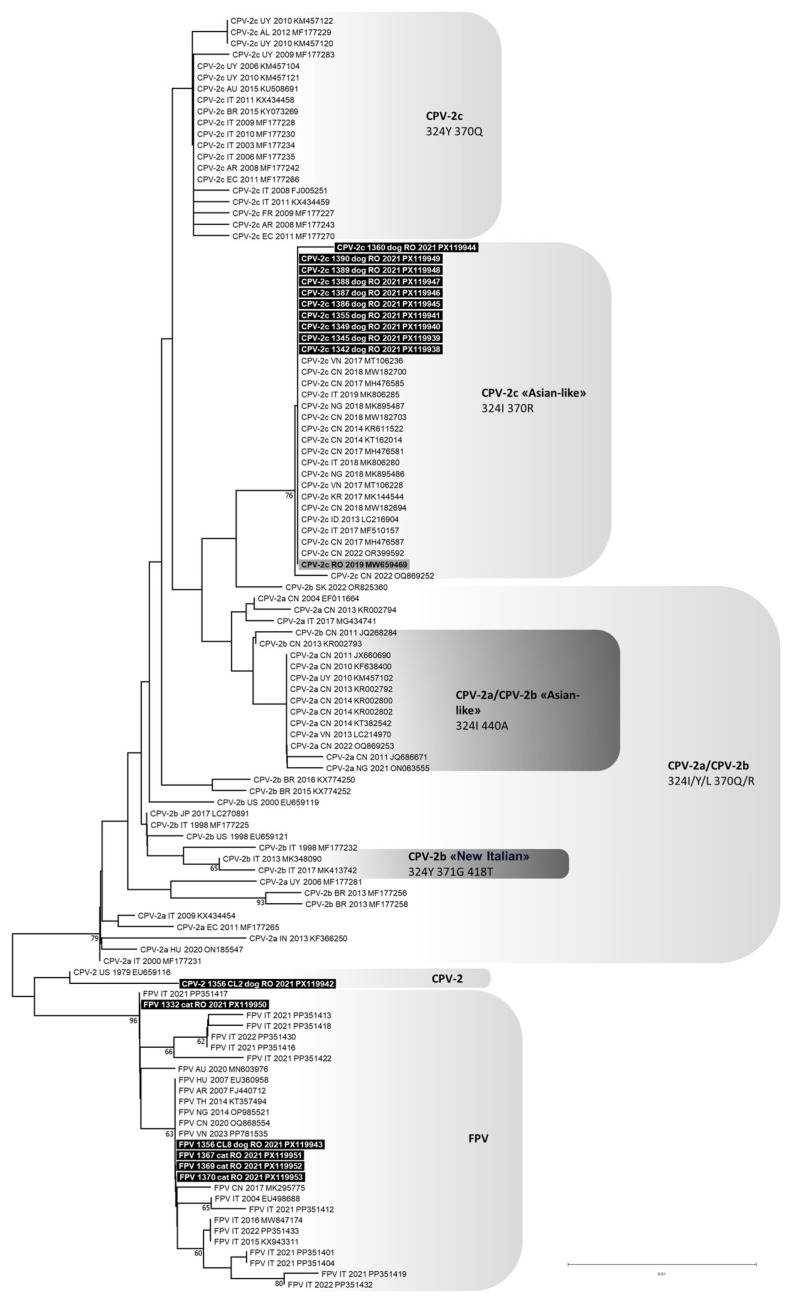
Phylogenetic tree constructed from the partial VP2 nucleotide sequences (532 nucleotides from positions 3669 to 4200 of FPV reference strain CU-4, GenBank ID: M38246) of *Protoparvovirus carnivoran 1* (PPVC-1) obtained in this study and reference strains retrieved from the GenBank database, using the Neighbour-Joining method and Tamura 3-parameter model with gamma distribution. Bootstrap values calculated on 1000 replicates and ≥60% are indicated on the respective branches. Highlighted in black: sequences of canine parvovirus type 2 (CPV-2) and feline panleukopenia virus (FPV) generated in this study (only one of the two identical CPV-2 clones is reported). Highlighted in grey: sequences generated in a previous study published by the same authors (only the sequence with GenBank ID: MW659469 is reported because all the sequences obtained were identical) [11]. To the right of the figure, the groups evidenced in this study are indicated.

RDP analyses predicted no potential recombination events for any of the viral sequences generated in this study. Phylogeny showed that all CPV-2c identified in this study clustered to the “Asian-like” CPV-2c clade, composed of CPV-2c strains detected in Asia, Africa and Europe, and characterized by a typical VP2 amino acid profile (Figure 1). The original CPV-2 type identified in two clones from dog 1356/2021 clustered with, but was distant from, the American reference strain type 2 (GenBank ID: EU659116). The FPV identified in cats and in one dog grouped with FPV reference strains detected in cats worldwide (Figure 1).

**Figure 2:**
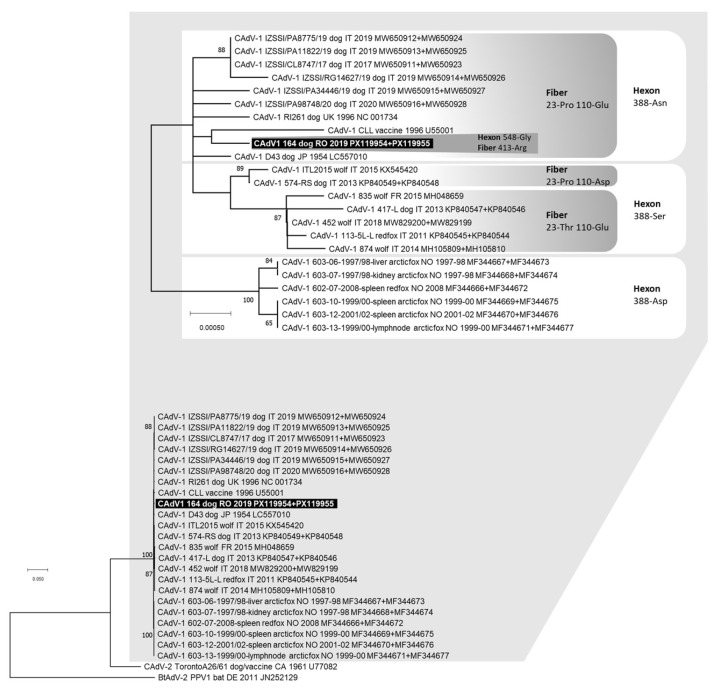
Phylogenetic tree constructed with the multiple gene approach (concatenated nucleotide sequences of the complete *hexon* and *fiber* genes) of Canine adenovirus (CAdV) obtained in this study and reference strains retrieved from the GenBank database, using the Maximum Likelihood method and Hasegawa–Kishino–Yano (HKY) model with gamma distribution and invariable sites. Bootstrap values calculated on 1000 replicates and ≥60% are indicated on the respective branches. On top of the figure, a portion of the obtained tree is enlarged to better visualize the phylogenetic relationships existing between the CAdV-1 nucleotide sequences and the bootstrap values. For some viruses, two GenBank accession numbers are reported (the *hexon* and *fiber* gene sequences, respectively). Highlighted in black: nucleotide sequence generated in this study. The amino acid residues in position 548 for the deduced hexon protein and in position 413 for the deduced fiber protein for sample 164/2019 are reported. The amino acid residues in position 388 for the deduced hexon protein and in positions 23 and 110 for the deduced fiber protein are also reported.

Complete nucleotide sequences of the CAdV-1 *hexon* (2718 nts) and *fiber* (1632 nts) genes were obtained from one dog (lab ID: 164/2019). The identified CAdV-1 exhibited two distinctive amino acid residues in the deduced hexon protein: 388-asparagine (Asn) and 548-glycine (Gly). Additionally, the deduced fiber protein had three distinctive amino acid residues: 23-proline (Pro), 110-glutamate (Glu) and 413-arginine (Arg). No end-point PCR products were obtained for the CAdV-2 detected by qPCR in the same dog. In the phylogenetic tree constructed from the concatenated nucleotide sequences of the *hexon* and *fiber* genes, CAdV-1 164/2019 clustered closely with the vaccine strain CLL and other CAdV-1 reference strains identified in dogs from Europe and Asia over 30 years ago. It also grouped with viruses recently detected in dogs from Southern Italy (Sicily, GenBank ID: MW650911–MW650928) (Figure 2).

**Figure 3:**
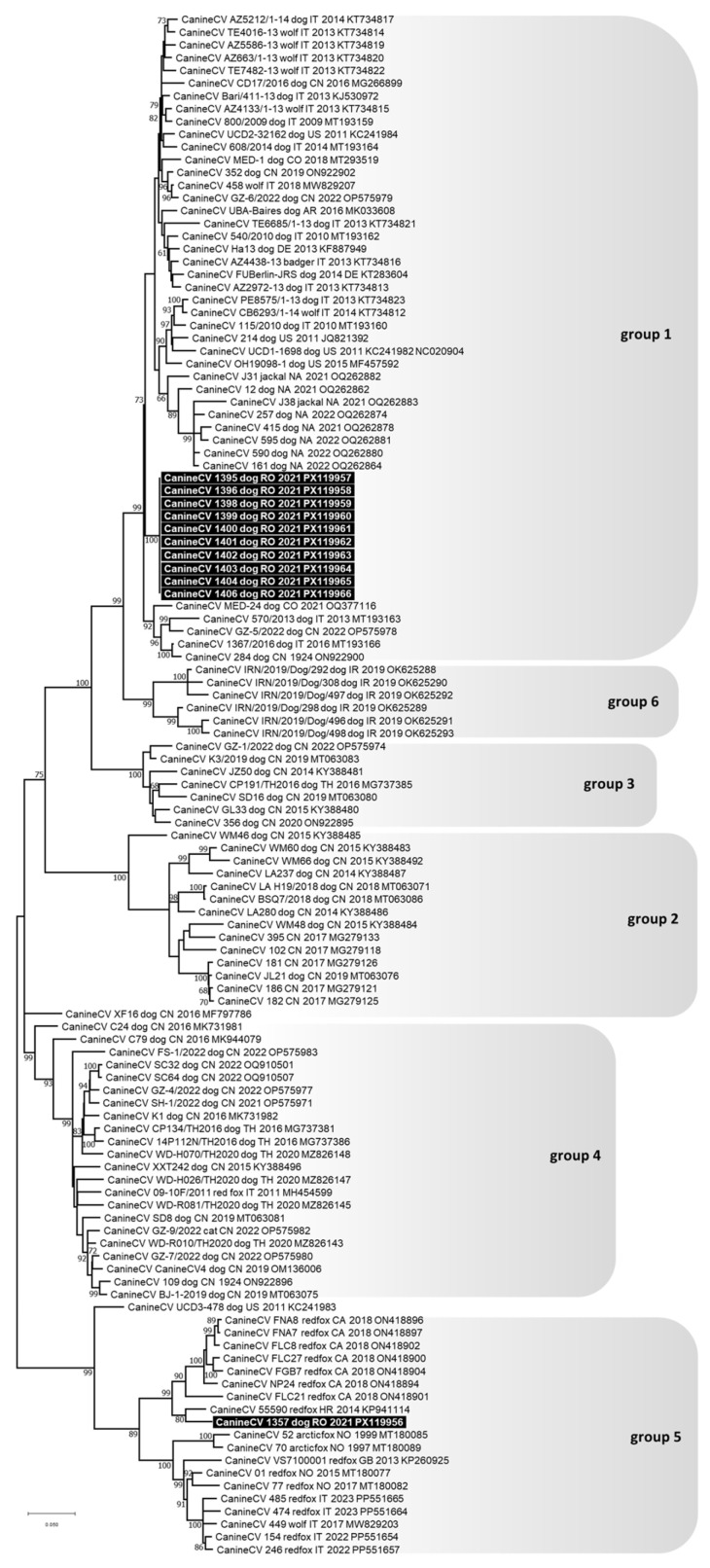
Phylogenetic tree constructed from the complete genome nucleotide sequences of Canine circovirus (CanineCV) obtained in this study and reference strains retrieved from the GenBank database, using the Maximum Likelihood method and General Time Reversible (GTR) model with gamma distribution and invariable sites. Bootstrap values calculated on 1000 replicates and ≥60% are indicated on the respective branches. Highlighted in black: sequences of CanineCV generated in this study. To the right of the figure, the groups evidenced in this study are indicated and correspond to the clusters proposed by Niu et al. [31], Urbani et al. [37], Beikpour et al. [45] and da Silva et al. [30].

The complete genomes of ten CanineCV detected in dogs were sequenced; they were 2063 nts in length, had complete nucleotide identity between each other and clustered in phylogenetic group 1, which included viruses that typically infect domestic dogs and wild carnivores (lab IDs: 1395/2021, 1396/2021, 1398/2021, 1399/2021, 1400/2021, 1401/2021, 1402/2021, 1403/2021, 1404/2021, and 1406/2021) (Figure 3). For another CanineCV, only a fragment of 809 nts in length was sequenced (from nt 652 to 1460 of the CanineCV reference strain 214, GenBank ID: JQ821392), and it clustered in phylogenetic group 5, which included viruses that primarily infect foxes but are also reported in wolves (lab IDs: 1357/2021) (Figure 3).

## 4. Discussion

This study reports an overall detection frequency of the investigated DNA viruses of 40/56 (71.4%) and 5/33 (15.2%) in dogs and cats, respectively, sampled in Romania between 2019 and 2021. These detection frequencies refer to PPVC-1, CAdV and CanineCV DNA in dogs and were significantly associated with age, vaccination status and the clinical presentation of the animals included in the study. A higher frequency of DNA virus infection, mainly due to CPV infection, was detected in young dogs with an incomplete vaccination status and clinical signs, consistent with available literature [46]. In contrast, only PPVC-1 DNA was detected in cats, and no associations were identified with animal data, probably due to the small size of the included population, which consisted exclusively of European shorthaired stray cats that were mostly adults and improperly vaccinated.

PPVC-1 DNA was detected in 36/56 (64.3%) dogs and in 5/33 (15.2%) cats. All but one of the PPVC-1 identified in dogs belonged to the CPV-2c variant, with amino acid residues in the deduced VP2 protein (324Ile and 370Arg) that are distinctive for Asian-like CPV-2c strains. These viruses were phylogenetically grouped with viruses from Asia [47,48,49,50,51], Italy [52,53] and Nigeria [54]. Notably, ten of the Asian-like CPV-2c identified in these dogs from Romania were previously reported by the same authors [11]. Assuming that Asian-like CPV-2c were introduced into Romania from Asia, the data presented in this study suggest a high level of diffusion across the territory, to the detriment of other CPV variants.

In the literature it has been hypothesized that Asian-like CPV-2c strains are more virulent compared to other CPV variants [51], due to amino acid mutations in the viral VP2 protein. In particular, the amino acid 324Ile may lead to stronger receptor binding [55,56], as it is adjacent to residue 323, which is involved in transferrin receptor binding and host-range mutation of the virus [57,58]. Differently, the amino acid 370Arg could be involved in a conformational change in the viral VP2 protein that is required during the replication cycle, or it may influence receptor binding through neighboring residues [51]. Residue 370 is conformationally adjacent to residues 379 and 384, which affect canine transferrin receptor binding and determine the canine host range [59]. Moreover, residue 370 is also close to residues 375 and 377, both associated with the ability of CPV to hemagglutinate or alter the pH dependence of hemagglutination [56,59].

In dog 1356/2021, a two-year-old male with an unknown vaccination status, a co-infection with CPV-2 and FPV was detected. The identified CPV-2 showed distinctive amino acid residues in the deduced VP2 protein: 375Asn, 386Lys and 418Thr. Mutation at residue 375 was shared with the American reference strain type 2 (GenBank ID: EU659116) [58] and was previously reported in CPV-2b strains [60]. In contrast, mutations at residues 386 and 418 were not shared with the American reference strain type 2 (GenBank ID: EU659116) [58]. The 386Lys mutation was introduced to attenuate a specific live-virus vaccine strain and was patented by the manufacturer [61], but it was also reported in vaccine strains before patent submission [62]. The 418Thr mutation, located in the GH loop of VP2, has been previously reported in Italy [62] and in several Asian countries, where it was commonly found in Korean and Japanese strains [63,64]. The FPV identified in dog 1356/2021, as well as in all but one of the cats in this study, was identical in the genetic trait analyzed, suggesting the predominant spread of a single strain in cats in Romania.

In the literature, CPV-2c has been reported in association with severe disease in dogs vaccinated with old type CPV-2-based vaccine [65]. In this study, Asian-like CPV-2c DNA was identified in fifteen dogs that had been previously vaccinated with the same category of vaccines (CPV-2-based) [11]. Additionally, a study conducted in China raised concerns about the efficacy of currently available vaccines in providing adequate protection against Asian-like CPV-2c infection in dogs [51]. Based on these observations, future studies should focus on evaluating the ability of currently used vaccines to prevent clinical manifestation and reduce the spread of Asian-like CPV-2c in domestic dog populations.

One dog (lab ID: 164/2019) tested positive for both CAdV-1 and CAdV-2 DNA (1/56, 1.8%). This dog, a four-month-old owned female Siberian Husky with an incomplete vaccination protocol (one dose of modified live CPV-2 vaccine), showed acute gastroenteritis and also tested positive for CPV-2 DNA [11]. Few studies have used molecular methods to investigate the simultaneous presence of CAdV-1 and CAdV-2 infections in dogs in Europe. In particular, the results of two studies conducted in Italy by Balboni et al. showed that 3.6% [66] and 7.8% [18] of dogs tested positive for both viral types, suggesting that co-infection with these two viruses is a relatively common occurrence. The detection of CAdV-1 infection highlights the potential epidemiological role of certain Eastern European countries, namely Hungary and Romania, which are known as significant exporters of purebred dogs [67]. Analysis of the complete *hexon* and *fiber* genes of CAdV-1 from dog 164/2019 revealed two amino acid mutations never previously reported in the literature (548-glycine in the deduced hexon protein and 413-arginine in the deduced fiber protein), and a close genetic relationship to reference strains circulating thirty or more years ago in Europe and Asia, including the CLL vaccine strain deposited in GenBank in 1996. This suggests a potential temporal stability of CAdV-1 circulating in Romania, but further time-scaled phylogenetic studies are needed to confirm this hypothesis. Notably, the CAdV-1 from dog 164/2019 showed a close genetic relationship to CAdV-1 strains recently detected in dogs from Southern Italy [68]. These findings, obtained through advanced molecular analysis, raise further questions about the potential transboundary spread of the virus through dog trade. No end-point PCR products were obtained from the CAdV-2 detected by qPCR in the same dog, probably due to the low amount of viral DNA in the fecal sample.

CanineCV DNA was detected in 15/56 (26.8%) clinically healthy dogs, with a significantly higher frequency observed in shelter dogs compared to owned dogs. Furthermore, CanineCV DNA was not detected in dogs with clinical signs of acute gastroenteritis in this study. Variable prevalence values of CanineCV infection have been reported, depending on the presence and severity of clinical signs [34,36,69], and its role in the etiopathogenesis of gastroenteritis remains debated [30]. A comparable infection rate (28.3%) has been reported in Italian dogs with no history of gastrointestinal pathology [24], while a lower frequency (7.3%) was found in healthy dogs from Germany [25]. Overall, these findings highlight CanineCV asymptomatic carriage and raise question about its potential primary role in the etiopathogenesis of canine gastrointestinal disorders [70]. Our results not only confirm the presence of CanineCV in apparently healthy dogs but also show a higher positivity rate in shelter dogs, suggesting the epidemiological importance of factors such as shared contaminated environment, population density, hygiene measures and welfare standards. Indeed, high population density in potentially contaminated environments with insufficient hygiene measures could explain the increased viral circulation observed in shelters. Conversely, lower positivity rates in owned dogs likely reflect reduced viral spread due to epidemiology-based prevention (e.g., reduced exposure and improved sanitary conditions). Although CanineCV infection has been sporadically reported in cats [30], viral DNA was not detected in the feline population in this study. However, the small sample size does not allow us to exclude the possibility that CanineCV is circulating in cats in Romania. Genetic characterization of the ten circoviruses identified in this study revealed that they belong to a phylogenetic group typically associated with domestic dogs (and wild carnivores) and that they have a complete nucleotide identity, suggesting that the same viral strain predominantly circulates in the Romanian canine population. An exception was a CanineCV identified in dog 1357/2021, which grouped phylogenetically with viruses primarily infecting foxes (and wolves), suggesting potential transmission between wild animals and dogs at the interface of wild and domestic environments. Therefore, the existence of a phylogenetic group composed solely of fox-infecting or wildlife-infecting CanineCV strains can be excluded.

Twelve out of the 40 (30%) dogs were co-infected: eleven tested positive for both Asian-like CPV-2c and CanineCV, while one was positive for Asian-like CPV-2c [11], CAdV-1 and CAdV-2. In the literature, CPV is frequently found in association with CAdV-1 and CAdV-2, which in some cases leads to a worsening of clinical signs and an increased mortality rate [66,67,71]. The association between CPV and CanineCV has also been reported in domestic dogs [35,36,66,72]. A synergistic effect of CanineCV infection has been hypothesized, allowing clinical disease to develop in CPV-infected dogs despite vaccination [23,35]. However, in our study, most of the dogs coinfected with CPV and CanineCV (8/11; 72.7%) were vaccinated young animals with no clinical signs. These findings align with previous reports that found no exacerbation of clinical conditions in dogs co-infected with CPV and CanineCV [73].

The main limitations of this study are related to its retrospective design, which affected both the size and composition of the study population. When interpreting our results, it should be noted that the samples were collected for convenience from a limited number of animals, either presenting clinical signs typically associated with gastroenteric infections or clinically healthy. Although these inclusion criteria did not allow for the analysis of an adequately sized study population to reliably assess the prevalence of the investigated viruses, they did enable us to demonstrate viral circulation and genetically characterize the identified pathogens. Additionally, the use of surplus clinical samples meant that we could not standardize the freeze–thaw cycles, or the amount of feces subjected to DNA extraction, which may have affected the sensitivity of viral DNA detection (potentially leading to underestimation of positive cases) and the comparison of detected viral DNA quantities. This limitation highlights the importance of conducting further prospective studies with larger, randomly selected populations, and standardized sample collection to better assess the epidemiological situation in Romania.

## 5. Conclusions

In this study carried out in Romania, CPV and CanineCV in dogs, as well as FPV in cats, were frequently detected in fecal samples using molecular methods. Additionally, CAdV-1 and CAdV-2 were identified in one dog co-infected with CPV. The detection of these DNA viruses was not consistently associated with clinical signs, stressing the importance of screening both symptomatic and asymptomatic domestic carnivores to assess viral circulation and implement effective control measures aimed at limiting the spread of infectious agents in susceptible animals. This study provides new data on the diffusion of Asian-like CPV-2c among dogs in Central Europe and raises concerns about the potential impact this virus may have on the health of the canine populations. Given current epidemiological trends for Asian-like CPV-2c, further investigations into the protective efficacy of existing preventive measures and tools (namely commercial vaccines) should be a priority. The first data on the presence of CAdV and CanineCV infection in dogs from Romania are also reported here. Genetic analysis of the identified CAdV-1 suggests potential temporal stability, and a close relationship with CAdV-1 strains recently detected in dogs from Southern Italy indicates possible spread through animal trade. Furthermore, genetically distinct CanineCVs were identified exclusively in healthy dogs, mainly from shelters, and frequently in co-infection with CPV. This suggests possible interspecies transmission from wild animals to domestic dogs, as well as greater viral diffusion within animal communities. Further studies are needed to clarify the role of CanineCV in the pathogenesis of enteropathies. When interpreted in light of potentially limited vaccination coverage and ongoing companion animal trade practices, the findings of this study emphasize the importance of sustained pathogen monitoring and strengthened preventive measures to mitigate the risk of infectious disease dissemination.

## Figures and Tables

**Table 1:** Dogs and cats included in this study and tested for *Protoparvovirus carnivoran 1*, Canine adenovirus and Canine circovirus DNA.

	Total	PPVC-1	*p* Value	CAdV	*p* Value	CanineCV	*p* Value	Total of Positive Animals ^3^	*p* Value
**Dogs**	56	36/56 (64.3)		1/56 (1.8)		15/56 (26.8)		40/56 (71.4)	
Sex									
Male	33/56 (58.9)	21/33 (63.6)	1	0/33 (0)	0.4107	9/33 (27.3)	1	22/33 (66.7)	0.3851
Female	23/56 (41.1)	15/23 (65.2)		1/23 (4.3)		6/23 (26.1)		18/23 (78.3)	
Age ^1^									
Young < 12 months	29/56 (51.8)	23/29 (79.3)	**0.0249**	1/29 (3.5)	1	10/29 (34.5)	0.2329	25/29 (86.2)	**0.0173**
Adult ≥ 12 months	27/56 (48.2)	13/27 (48.1)		0/27 (0)		5/27 (18.5)		15/27 (55.6)	
Breed									
Breed	4/56 (7.1)	4/4 (100)	0.2853	1/4 (25)	0.0714	0/4 (0)	0.5646	4/4 (100)	0.3148
Mixed breed	52/56 (92.9)	32/52 (61.5)		0/52 (0)		15/52 (28.8)		36/52 (69.2)	
Habitat									
Owned dogs	30/56 (53.6)	18/30 (60)	0.5796	1/30 (3.3)	1	1/30 (3.3)	**0.0001**	19/30 (63.3)	0.2357
Private shelter	26/56 (46.4)	18/26 (69.2)		0/26 (0)		14/26 (53.8)		21/26 (80.8)	
Vaccination									
Yes	31/56 (55.3)	14/31 (45.2)	**0.0017**	0/31 (0)	0.4364	11/31 (35.5)	0.1407	17/31 (54.8)	**0.0031**
No or incomplete	24/56 (42.9)	21/24 (87.5)		1/24 (4.2)		4/24 (16.7)		22/24 (91.7)	
NA	1/56 (1.8)	1/1 (100)		0/1 (0)		0/1 (0)		1/1 (100)	
Clinical status									
Clinically healthy	41/56 (73.2)	21/41 (51.2)	**0.0004**	0/41 (0)	0.2679	15/41 (36.6)	**0.0054**	25/41 (61)	**0.0028**
Acute gastroenteritis	15/56 (26.8)	15/15 (100)		1/15 (6.7)		0/15 (0)		15/15 (100)	
**Cats**	33	5/33 (15.2)		NT		0/33 (0)		5/33 (15.2)	
Sex									
Male	5/33 (15.2)	1/5 (20)	1	-	-	0/5 (0)	NT	1/5 (20)	1
Female	28/33 (84.8)	4/28 (14.3)		-		0/28 (0)		4/28 (14.3)	
Age ^1^									
Young < 12 months	5/33 (15.1)	1/5 (20)	1	-	-	0/5 (0)	NT	1/5 (20)	1
Adult ≥ 12 months	28/33 (84.9)	4/28 (14.3)		-		0/28 (0)		4/28 (14.3)	
Breed									
European shorthair	33/33 (100)	5/33 (15.2)	-	-	-	0/33 (0)	-	5/33 (15.2)	-
Habitat									
Stray cats	33/33 (100)	5/33 (15.2)	-	-	-	0/33 (0)	-	5/33 (15.2)	-
Vaccination									
Yes	9/33 (27.3)	2/9 (22.2)	0.5970	-	-	0/9 (0)	NT	2/9 (22.2)	0.5970
No or incomplete	24/33 (72.7)	3/24 (12.5)		-		0/24 (0)		3/24 (12.5)	
Clinical status									
Clinically healthy	26/33 (78.8)	3/26 (11.5)	0.2819	-	-	0/26 (0)	NT	3/26 (11.5)	0.2819
Acute gastroenteritis or other ^2^	7/33 (21.2)	2/7 (28.6)		-		0/7 (0)		2/7 (28.6)	

Data in brackets are expressed in %. *p* value < 0.05 was considered significant (bold). Unavailable data were excluded from the statistical analysis. ^1^ Data are reported as median and (range minimum-maximum). ^2^ Respiratory signs (*n* = 2 stray cats), pyometra (*n* = 1 stray cat), feline infectious peritonitis (*n* = 1 stray cat). ^3^ Total of positive animals to one or more of the pathogens screened. CAdV: Canine adenovirus; CanineCV: Canine circovirus; NA: not available; NT: not tested; PPVC-1: *Protoparvovirus carnivoran 1*.

## Data Availability

All data generated or analyzed during this study are included in this published article. The original data presented in the study are openly available in AMSActa UNIBO at [https://doi.org/10.6092/unibo/amsacta/8560 (accessed on 21 October 2025)]. The nucleotide sequences generated and analyzed during the current study are available in the International Nucleotide Sequence Database Collaboration repository (INSDC, http://www.insdc.org/, accessed on 13 August 2025) with the IDs: XP119938-XP119966.
